# Precision Cardio-Oncology: A Multimodal Approach to Cancer Therapy-Related Cardiovascular Toxicity Prediction and Assessment

**DOI:** 10.3390/cancers18203271

**Published:** 2026-10-10

**Authors:** Carl Simela, Ishikaa Mukherjee, Abhinav Kandala, Aruni Ghose, Turja Chakraborty, Navin Mathiyalagan, Amitava Banerjee, Arjun K. Ghosh

**Affiliations:** 1 Cardio-Oncology Service, University College London Hospitals NHS Foundation Trust, London NW1 2PG, UK; 2 Hatter Cardiovascular Institute, University College London, London WC1E 6HX, UK; 3 Barts and The London School of Medicine and Dentistry, Queen Mary University of London, London E1 2AD, UK; 4 Faculty of Medical Sciences, University College London, London WC1E 6BT, UK; 5 Velindre Cancer Centre, Velindre University NHS Trust, Cardiff CF14 2TL, UK; 6 Global Business School for Health, Faculty of Population Health Sciences, University College London, London E20 2AE, UK; 7 Department of Cardiology, London North West University Healthcare NHS Trust, London HA1 3UJ, UK; 8 National Heart and Lung Institute, Faculty of Medicine, Imperial College London, London SW3 6LY, UK; 9 Oncology and Cardio-Oncology Services, Nottingham University Hospitals NHS Trust, Nottingham NG7 2UH, UK; 10 Institute of Health Informatics, Faculty of Population Health Sciences, University College London, London NW1 2DA, UK; 11 Cardio-Oncology Service, Barts Heart Centre, Barts Health NHS Trust, London EC1A 7BE, UK; 12 William Harvey Research Institute, NIHR Barts Biomedical Research Centre, Faculty of Medicine and Dentistry, Queen Mary University of London, London EC1M 6BQ, UK

**Keywords:** precision, multi-omics, multimodality imaging, risk prediction models

## Abstract

Cancer therapy-related cardiovascular toxicity (CTR-CVT) is associated with morbidity, mortality, and interruptions to cancer treatment. Current cardio-oncology clinical assessment tools generally rely on a limited range of clinical data; however, in the era of precision medicine, it may be possible to leverage multimodal data, including imaging and multi-omic inputs, to personalise CTR-CVT prediction and assessment. In this review, we examine the genotypic and phenotypic predictors of CTR-CVT and the evidence supporting their incorporation into multimodal risk models. Most research focuses on cancer therapy-related cardiac dysfunction (CTRCD). We identify a strong evidence base for multimodal imaging-guided prediction, particularly cardiovascular magnetic resonance (CMR) and transthoracic echocardiography (TTE)-derived global longitudinal strain measurements. Emerging evidence also supports the potential value of novel imaging parameters, including computed tomography (CT) and TTE-derived radiomic features. From the genomic and proteomic perspective, truncating titin variants and microRNA-126 may be informative predictors of cancer therapy-related cardiac dysfunction (CTRCD). At the same time, the literature also highlights potential epigenomic markers of CTR-CVT. Finally, we consider how these diverse data can be integrated into multimodal risk models and how digital innovation and advanced machine learning methods may further improve model predictive performance. However, a large implementation gap remains, with challenges such as data privacy and a lack of external validation studies preventing routine clinical implementation.

## Simple Summary

Cancer treatments can cause cardiovascular toxicity, and research shows cancer survivors have an increased risk of cardiovascular disease. However, risk varies from person to person, so research efforts have focused on developing new methods to predict cancer therapy-related cardiovascular toxicity (CTR-CVT) to inform cancer treatment planning and guide initiation of cardioprotective treatment. The growth of novel scientific fields, such as multi-omics (the comprehensive assessment of multiple biological molecules) and radiomics (the assessment of quantitative features, such as pixel intensity, embedded in medical images), offers a potential way to individualise CTR-CVT prediction and assessment further, enabling more robust cancer treatment decisions. In this review, we consider the evidence supporting a multimodal approach to CTR-CVT assessment and prediction.

## 1. Introduction

Given its associated morbidity and mortality and its potential to interrupt lifesaving cancer treatment, cancer therapy-related cardiovascular toxicity (CTR-CVT) warrants early recognition [1,2]. CTR-CVT encompasses cancer therapy-related cardiac dysfunction (CTRCD) and other toxicities that can occur soon after cancer treatment begins or several decades later [3]. Substantial efforts have focused on devising methods to predict, assess, and risk stratify CTR-CVT to facilitate primary prevention and cardiovascular surveillance both during treatment and in the late effects setting [4,5,6,7]. Existing multivariable CTR-CVT risk prediction models apply predictor variables that are readily available in clinical practice, such as comorbidity status and demographic information, with varying discriminative power [8]. A genotype–phenotype integrated multimodal approach leveraging multiple individual-specific predictors, such as genetics and imaging data, as well as comprehensive molecular assessment data (multi-omics), may yield further predictive precision. This is particularly true with the expanding applications of artificial intelligence (AI) [9,10]. This personalised precision prediction of CTR-CVT aims to move cardio-oncology from static and standardised surveillance towards dynamic and individualised prevention, monitoring and treatment [11].

Here, we present a narrative review of the literature on phenotypic and genotypic predictors of CTR-CVT, as well as novel multimodal CTR-CVT prediction models. We also discuss how digital innovation could contribute to CTR-CVT risk prediction. This narrative review summarises and critically appraises the literature, while providing insights for precision cardio-oncology and CTR-CVT prediction.

## 2. The Phenotype Approach: Multimodal Imaging

There is growing evidence supporting the role of multimodal imaging for predicting and assessing CTR-CVT, including but not limited to CTRCD (Table 1) [12,13,14].

### 2.1. Computed Tomography

Cardiac computed tomography (CT), including coronary CT angiography (CCTA), has a role in CTR-CVT prediction [15] (Table 1). Large cohort studies have well established the predictive value of coronary artery calcium (CAC) scoring and CCTA for coronary artery disease (CAD). More recently, these methods have been studied in the context of CAD associated with cancer treatment [12,16]. Beyond this, there is increasing evidence that pre-treatment CAC may predict anthracycline-induced CTRCD [17,18] and major adverse cardiovascular events [18], but this is yet to be implemented in routine clinical practice. CAC scores can also be obtained from non-gated chest CT scans, which highlights the potential for opportunistic CTR-CVT risk stratification during routine cancer assessment and staging [18]. Other studies have implicated baseline CT-derived fractional flow reserve and CT-measured portal phase myocardial extracellular volume as potential novel predictors of subsequent CTRCD in patients with breast cancer receiving anthracyclines [19,20]. Early experimental data also suggests prediction models incorporating CT radiomic features may be useful, particularly those based on the Gray-Level Run Length Matrix [21,22].

### 2.2. Cardiovascular Magnetic Resonance

Cardiovascular magnetic resonance (CMR) is the gold-standard non-invasive method for assessing both cardiac function and myocardial tissue, giving it an important role in CTR-CVT prediction and assessment [23,24]. As with echocardiography-derived global longitudinal strain (GLS), evidence supports CMR-derived GLS as an early marker of CTRCD [25]. The literature is conflicting as to the role of CMR tissue biomarkers, including late gadolinium enhancement (LGE), for predicting CTRCD, with several studies finding either no role or a limited role [26,27,28,29] and others finding a stronger role [30,31]. The percentage of normal myocardium segments at baseline, as assessed by circumferential and longitudinal strain, may be another potential CMR-derived predictor, as one study reported an area under the curve (AUC) of 0.76 for predicting symptomatic CTRCD [32]. CMR radiomic features are also relatively unexplored in the CTR-CVT setting.

### 2.3. Echocardiography

Echocardiography is the cornerstone of CTRCD assessment and surveillance. As reflected in international guidelines, left ventricular GLS has the most robust evidence base for early prediction of LVEF decline of all widely used clinical echocardiographic parameters [2,33]. However, clinical trials assessing a GLS-guided approach for cardioprotective therapy initiation have generally not shown clear superiority over a LVEF-guided approach [34]. Other echocardiography-derived early markers of CTR-CVT include right ventricular strain measurements and volume measurements [35,36] (Table 1). Three-dimensional echocardiography also offers pulse wave velocity increases and longitudinal strain reductions during chemotherapy as potential early markers of CTRCD [37]. A recent study suggested early diagnosis of CTRCD may be further improved by combining individual parameters, such as 3D LVEF, 2D GLS and 2D global circumferential strain, during echocardiographic surveillance of women with early-stage breast cancer [38]. Lastly, machine learning models comprising pre-chemotherapy echocardiographic radiomic input features may have a role in CTRCD prediction, as suggested by a recent study [39].

### 2.4. Nuclear Imaging

Nuclear imaging techniques, including Positron Emission Tomography (PET) and Multigated Acquisition (MUGA), may be useful for CTR-CVT prediction [40]. Single Photon Emission Tomography (SPECT) has an established role in myocardial perfusion imaging and ischaemic assessments, which can be applied to the cardio-oncology setting [41]. Traditionally, MUGA was used for diagnosing CTRCD [42]. However, echocardiography and CMR have since superseded MUGA, as recent studies have highlighted its poor sensitivity for CTRCD diagnosis and the poor accuracy of MUGA-acquired LVEF measurements compared with echo and CMR references, particularly in individuals with smaller indexed left ventricular end-diastolic volumes [43,44,45]. Therefore, MUGA remains useful only when quality imaging with CMR or echocardiography is infeasible or contraindicated.

Some studies have suggested that fludeoxyglucose F18 (^18^F-FDG) uptake, quantified by standardised uptake value (SUV), can predict CTR-CVT. For example, Bauckneht and colleagues [46] implicated low left ventricular (LV) SUV before cancer treatment as a predictive marker of cardiac abnormalities, including LVEF decline and diastolic dysfunction, following doxorubicin treatment for Hodgkin’s lymphoma [46,47]. However, this study was limited by a small sample of 36 participants and only two cases of LVEF decline, which limits the inferences that can be drawn. Larger studies have found similar positive findings, including a recent study that found an association between CTR-CVT and abnormal ^18^F-FDG uptake pattern changes after anthracycline in a cohort of 366 lymphoma patients [48]. Of note, however, this was a single-centre study, and an abnormal 18F-FDG uptake pattern change included uptake that was initially normal and then became abnormal after six cycles of chemotherapy. It is unclear whether the abnormal 18F-FDG uptake occurs early enough to allow initiation of prognostic cardioprotective therapy, particularly as another study found that SUV had predictive power for end-of-treatment LVEF only after two cycles of anthracyclines [49]. Taken together, these findings suggest that PET-CT ^18^F-FDG uptake measures may be more useful for identifying early subclinical CTRCD than for predicting cardiovascular toxicity before cancer treatment begins. However, this needs further research, as a prospective multicentre study found no association between subclinical CTRCD, diagnosed by a decrease in GLS, and SUV measured before, during, or after anthracycline chemotherapy [50].

**Table 1:** Available and emerging multimodal imaging predictors and markers of cancer therapy-related cardiovascular toxicity from recent human studies.

Imaging Modality	Imaging Variable/Marker	Key Reference(s)	Prediction/Association
Computed tomography	Coronary artery calcium score	[16]	Predicts coronary artery disease
		[17,18]	Positive association with anthracycline-induced CTRCD
	Fractional flow reserve	[19]	
	Myocardial extracellular volume	[20]	
	Radiomic features (GLNUN, LDHGLE, SRHGLE, RLNN, HGLE)	[51]	Predicted chemoradiation-induced heart failure in breast cancer
Cardiovascular magnetic resonance	Extracellular Volume	[26]	Associated with concurrent and subsequent CTRCD
	T2 relaxation time	[31]	Increased T2 predicted anthracycline-induced LVEF decline
	T1 relaxation time	[30]	Increased T1 predicted anthracycline-induced LVEF decline
	LV GLS	[25]	Predicted subsequent anthracycline-induced CTRCD
	Normal myocardium %	[32]	
Echocardiography	LV GLS	[33,52,53]	Decline in these parameters after anthracycline predicted CTRCD (defined as a drop in LVEF)
	LVESV	[52]	
	LVEDV	[52]	
	LV GCS	[38]	Associated with CMR-defined CTRCD
	Global work index and global constructive work	[54]	
	RVLFS	[55]	RVLFS decrease > 12.4% and RVESV increase > 12.2% each predicted subsequent RVEF decline after anthracycline
	RVESV		
	Pulse wave velocity	[37]	Increase in pulse wave velocity after the third cycle of anthracycline-containing chemotherapy predicted subsequent LVEF decline
	Radiomic features (Wavelet, LBP, HOG, Tamura, Frequency, SFTA, Laws, shape and Haralick)	[39]	Pre-anthracycline/HER2 2D radiomic features predicted chemotherapy-induced LVEF decline
PET/Nuclear imaging	^18^F-FDG uptake or SUV	[46]	Low baseline * LV SUV predicted cardiac abnormalities after doxorubicin, including LVEF decline and AF
		[48]	Abnormal ^18^F-FDG uptake pattern change post-anthracycline predicted CTR-CVT
		[49]	Higher interim * and end-of-treatment LV SUV associated with LVEF decline after anthracycline

AF atrial fibrillation; EDV end diastolic volume; ESV end systolic volume; HGLE High Gray-Level Emphasis; GCS global circumferential strain; GLNUN Gray-Level Non-Uniformity Normalized; GLS global longitudinal strain; LDHGLE Large Dependence High Gray-Level Emphasis; LFS lateral free wall strain; LV left ventricle; LVEF left ventricular ejection fraction; PET positron emission tomography; RLNN Run-Length Non-Uniformity Normalized; RV right ventricle; SRHGLE Short Runs High Gray-Level Emphasis; SUV standardised uptake value; * baseline refers to before cancer treatment and interim refers to during cancer treatment.

## 3. The Genotype Approach

### 3.1. Genomics

Researchers have used candidate gene analyses and genome-wide association studies (GWAS) to investigate genetic markers of CTR-CVT (Table 2). Chaix et al. [56] identified 31 genes with a lower burden of rare variants among childhood cancer survivors who developed CTRCD compared to those who did not. In most cases, the rare variants were missense, and in two cases, in vitro inhibition of the associated gene pathways (*ZNF827* and *PI3KR2)* protected against doxorubicin-mediated cardiotoxicity. Conversely, substantial evidence links a high burden of rare truncating variants in titin (*TTN*) to cancer therapy-induced cardiomyopathy (CCM) [57,58,59]. For example, one study found a 7.5% prevalence of *TTN* truncating variants in patients with CCM compared to a 0.7% prevalence in healthy volunteers and a 0.9% prevalence in an unselected breast cancer cohort [60]. *TTN* truncating variants have also been implicated in familial and idiopathic dilated cardiomyopathy (DCM) [61]. Other work has focused on other DCM genes, such as the troponin-I interacting kinase (*TNNI3K*) and ryanodine receptor 2 (*RYR2*), with Mahmoud et al. (56) showing increased expression of both genes after anthracycline treatment in patients who developed CTRCD compared with those who did not. Another study found an association between rare missense variants in known idiopathic and familial DCM genes, including *RYR2* and lamin A/C (*LMNA),* in anthracycline-induced CTRCD but not in trastuzumab-induced CTRCD [57]. These findings have three implications. Firstly, this suggests that rare genetic variants can be protective or deleterious against CTRCD, and there may be a possible role for rare variants and gene expression levels as predictors of anthracycline-induced cardiac dysfunction; however, for the latter to be clinically useful, the increase in gene expression must precede clinical CTRCD by long enough to allow early initiation of prognostic cardioprotective therapy. Second, these results suggest anticancer treatment-type-based variation in the efficacy of a genetics-based risk prediction approach, with the most robust evidence base currently for anthracycline-induced CTRCD prediction. Lastly, the idiopathic and familial DCM genotype may be a substrate for anthracycline-induced CTRCD, which should be considered in precision cardio-oncology algorithms.

Beyond rare-variant analysis, GWAS and exome-wide association studies (ExWAS) have examined whether common genetic variants can predict CTR-CVT. Indeed, five genetic variants (*SLC28A3* rs7853758, *CELF4* rs1786814, *RARG* rs2229774, *UGT1A6* rs17863783 and rs28714259 intergenic variant) are cited in the 2022 European Society of Cardiology cardio-oncology guidelines as drivers of anthracycline-related CTRCD [2]. However, data from a CARDIOTOX registry analysis found that only the rs28714259 intergenic variant *p*-value approached the genome-wide significance threshold (*p* = 1 × 10^−3^) [62]. The same study implicated an intergenic variant on chromosome 3 (rs37652759) between the *TPRG1* and *TP63* genes as a novel CTRCD susceptibility locus, and subsequent chromatin interaction mapping identified five candidate genes (*KNG1, MASP1, TP63, LEPREL1* and *CLDN1*) possibly affected by this intergenic locus and therefore may have a role in CTRCD prediction. These results are particularly important because they come from a genome-wide meta-analysis, which increases power to detect genetic associations. Other potential genetic variants for CTR-CVT prediction include *SLC28A3*-rs7853758, *P2RX7*-rs208294 and *ABCC1*- rs45511401 as recently investigated in two systematic reviews (Table 2). Compared to other genetic variants, these findings have a stronger evidence base, having been validated in systematic reviews [63,64].

There may be further predictive power to be gleaned using logic regression, where predictors are formed from Boolean combinations of multiple single nucleotide polymorphisms (SNPs), or polygenic risk scores (PGSs), where multiple SNPs are combined to a single estimate of genetic variation contributing to a specific phenotype [65,66]. Sharafeldin et al. [67] used the former methodology to show that having no or only one copy of the rs208294 (T>C), rs3751143 (A>C), and rs3751144 (C>T) variant alleles protected against CTRCD. PGSs have been investigated for CTR-CVT prediction, with a recent study showing atrial fibrillation (AF), coronary artery disease (CAD), myocardial infarction (MI) and hypertension (HTN) PGSs predict a range of cardiovascular diseases in women with a history of breast cancer (Table 2) [68]. Bauer and colleagues also implicated epigenetic predictors of CTR-CVT, finding 185 differentially methylated regions between the genomes of doxorubicin-treated patients with breast cancer who developed cardiotoxicity and those who did not. Specifically, they found increased pre-treatment *IRF6* and *SNFN12* methylation, but reduced pre-treatment *RNF39* methylation was associated with LVEF decline after doxorubicin treatment [69]. In another study, methylation-based assessment of biological age was positively associated with LVEF decline after trastuzumab treatment [70]. Subsequent studies have built on this exploratory work to create risk prediction models incorporating both clinical and genetic input features, which in some cases improves predictive performance compared with models using clinical features alone [56,70,71]. However, external cohort validation is needed.

**Table 2:** A selection of possible genetic markers of cancer therapy-related cardiovascular dysfunction (CTR-CVT) and cardiovascular disease in people with cancer/cancer survivors.

Genetic Marker(s)	Reference(s)	Population(s)	Findings
*TNNI3K* *RYR2*	[72]	Women treated for breast cancer	Increased expression after anthracycline treatment was a marker of anthracycline-induced CTRCD in women treated for breast cancer
Polygenic scores: AF (PGS0003331) CAD (PGS003725) MI (PGS000710) HTN (PGS000900)	[68]	Women with a history of breast cancer	AF PGS predicted any CVD, serious CVD and arrhythmia CAD PGS predicted ischaemic heart disease MI PGS predicted ischaemic heart disease CAD and MI PGS combination predicted ischaemic heart disease HTN PGS predicted hypertension
*ZNF827* *PI3KR2* *ELAC2* ^1^	[56]	Paediatric cancer survivors who received anthracyclines	Lower burden of *ZNF827*, *PI3KR2* and *ELAC2* rare variants amongst patients that developed anthracycline cardiotoxicity compared to those that did not
*TTN*	[60]	Adults with diverse cancers and children with AML	Higher burden of *TTN* truncating variants amongst patients with CCM (7.5%) compared to healthy volunteers (0.7%), unselected breast cancer patients (0.9%) and unselected lung cancer patients (1.3%)
	[57,58,59]	Adults with haematological and breast malignancy	Higher burden of *TTN* truncating variants amongst patients with anthracycline-induced CCM
rs37652759 (C>T)	[62]	Adults with diverse cancers	Effect allele associated with CTRCD Chromatin interaction mapping identified *KNG1, MASP1, TP63, LEPREL1* and *CLDN1* as candidate genes
*P2RX7*	[63,67]	Childhood cancer survivors treated with anthracycline	Having no or only one copy of rs208294 (T>C), rs3751143 (A>C) and rs3751144 (C>T) variant alleles protected against CTRCD
*RARG*-rs2229774 (G>A)	[73]	Childhood cancer survivors	*RARG*-rs2229774 G>A allele associated with CTRCD
*SLC28A3*-rs7853758 (G>A)	[74]	Paediatric ALL and osteosarcoma survivors	*SLC28A3*-rs7853758 AA genotype associated with CTRCD
*CYBA*-rs4673 (A>G, T) *RAC2*-rs 13058338 (T>A, G) *CYP3A5*-rs776746 (T>C) *ABCC1*- rs45511401 (G>T) *ABCC2-* rs8187710 (G>A) *ERBB2*- rs1136201 (A>G, T)	[64]	Adults and children with variety of cancers	Variant alleles associated with CTR-CVT
*SLFN12, IRF6* and *RNF39* methylation	[69]	Adults with breast cancer treated with doxorubicin	Increased baseline *IRF6* and *SNFN12* methylation, but reduced *RNF39* methylation associated with reduced LVEF post-DOX
AgeAccel epigenetic clocks: Hannum, Horvath, Horvath Skin and Blood, PhenoAge, GrimAge, DunedinPACE metrics	[70]	Adults with breast cancer treated with trastuzumab	Hannum AgeAccel, Hovarth AgeAccel, and Horvath Skin and Blood AgeAccel epigenetic ages positively associated with LVEF decline in models adjusted for age, risk factors and blood cell composition

ALL acute lymphoblastic leukaemia; AML acute myeloid leukaemia; CAD coronary artery disease; CCM cancer therapy-induced cardiomyopathy; CVD cardiovascular disease; DOX doxorubicin; MI myocardial infarction; PGS polygenic score. ^1^ There were 28 other genes with differential enrichment but did not undergo functional testing.

### 3.2. Circulating Biomarkers and Proteomics

Research on circulating biomarkers for cardio-oncology risk prediction has primarily focused on CTRCD. Landmark papers from the late 1990s and early 2000s highlighted the value of cardiac troponins and natriuretic peptides for the prediction and early diagnosis of anthracycline-induced CTRCD, respectively [75,76]. These results have since been confirmed in several newer studies, including a meta-analysis of 23 studies showing a negative cardiac troponin result had a negative predictive value of 93% against LV systolic dysfunction following a range of chemotherapeutic agents, but natriuretic peptides had a less clear predictive role [77]. Because meta-analyses represent the pinnacle of the evidence hierarchy, these findings provide strong evidence for using cardiac troponins to predict CTRCD. Consequently, pivotal position papers from global cardiology societies advocate using troponin for CTRCD prediction and diagnosis [78,79].

Emerging evidence suggests well-established circulating biomarkers, such as C-reactive protein (CRP), could be repurposed for CTR-CVT prediction, whilst creatine kinase and lactate dehydrogenase may be elevated in immune checkpoint inhibitor-related myocarditis (ICIM); thus, measuring levels may facilitate early diagnosis [80,81]. However, these biomarkers often lack sufficient specificity to establish the ICIM diagnosis independently, as shown in one cohort where elevated creatine phosphokinase during immune checkpoint inhibitor therapy demonstrated 99% sensitivity but only 23% specificity [80]. Furthermore, universally validated ICIM-specific thresholds for troponin and natriuretic peptides have not been established, and prospective validation of biomarker-based surveillance strategies remains necessary. Table 3 shows some potential novel proteomic markers for CTR-CVT. Of these novel markers, the strongest evidence currently exists for myeloperoxidase (MPO) levels, with a contemporary meta-analysis finding that an increase in serum MPO levels after doxorubicin and trastuzumab conferred a 30% increased rate of CTRCD [82]. No such finding was observed for the other four biomarkers tested (CRP, growth differentiation factor-15, galectin-3, and placental growth factor). Also, a previous study found MPO predictive power was maintained across different cardiotoxicity definitions [83]. It also had a favourable kinetic profile compared with other prospective biomarkers, such as placental growth factor, which remains elevated for at least 15 months.

### 3.3. Transcriptomics

Transcriptomics may also have a role in CTR-CVT prediction and early detection [84]. Compared to other miRNAs, stronger evidence exists for miRNA 126, as it shows a strong positive correlation with cardiac troponin I and cardiac troponin T increases at six months after anthracycline treatment initiation [85]. This may be useful for early detection of CTRCD. miRNA 126 may also have a role in predicting CTRCD, as a recent systematic review pathway analysis found a negative association between baseline miRNA 126 expression levels and subsequent CTRCD following anthracycline treatment [86]. However, large multi-centre prospective studies with extended follow-up beyond 12 months that correlate transcriptomic signatures before and during cancer treatment with subsequent CTR-CVT are lacking.

**Table 3:** Possible proteomic and transcriptomic biomarkers of cancer therapy-related cardiovascular dysfunction (CTR-CVT) and cardiovascular disease in people with cancer/cancer survivors from recent human studies.

Circulating Biomarkers	Key Reference(s)	Summary of Findings
Cardiac Troponins	[53,75,77]	Predicts LV dysfunction and is an early marker of CTRCD with strong negative correlation with LV GLS
Natriuretic peptides	[53,77]	Early marker of subclinical CTRCD with moderate positive correlation with LV GLS. Less clear role for predicting LV dysfunction
C-reactive protein	[81]	Elevated CRP after 1st cycle of doxorubicin associated with subsequent LVEF decline
	[87]	Negative association between serum CRP and LVEF
Myeloperoxidase	[81,82,83,88,89]	Elevated baseline levels predict CTRCD in women with breast cancer treated with anthracyclines and/or trastuzumab
Matrix Metalloprotease 9	[85]	Correlation with established markers of CTRCD (troponin and NP)
MicroRNAs	[85,86,88]	Several microRNAs (including miR-4461, Let-7f, miR-20a, miR-1, miR-126 and miR-210), upregulated at baseline in patients with breast cancer treated with AC and/or trastuzumab that go on to develop CTRCD
sST2	[90]	Negative correlation between sST2 and LVEF during anthracycline and/or anti-HER2 treatment
MIF	[91]	Elevated levels at 2nd cycle of doxorubicin predicted LVEF decline after 4th cycle
PIGF	[83]	Increased PIGF predicted subsequent LVEF decline in women with breast cancer treated with anthracyclines and trastuzumab

CRP reactive protein; LVEF left ventricular ejection fraction; LV GLS left ventricular global longitudinal strain; MIF macrophage migration inhibitory factor; NP natriuretic peptides; PIGF placental growth factor; sST2 soluble suppression of tumorigenicity.

## 4. Experimental Multimodal Risk Models

Several risk scores have been developed to predict CTR-CVT. They generally use a limited range of clinical variables, including oncological data, patient demographic data and blood test results [92]. The Heart Failure Association and International Cardio-Oncology Society (HFA-ICOS) baseline cardiology risk assessment tools for several therapies, including human epidermal growth factor receptor 2 (HER2) therapies and anthracyclines, were among the first widely validated tools to use a multimodal approach for CTR-CVT risk assessment [5]. In addition to clinical variables, these tools incorporate left ventricular ejection fraction (LVEF) as an imaging measure and serum troponin and NT-proBNP as biomarker measures. In recent years, researchers have considered other modalities. For example, two recent studies have shown increased CTR-CVT model predictive performance by combining traditional clinical variables with novel genetic predictor variables in women with breast cancer and childhood cancer survivors [56,68]. Several other studies have also combined different types of data to stratify CTR-CVT risk (Table 4). One study found that logistic regression models combining serum biomarkers and echocardiographic predictors showed good CVD predictive performance (AUC: 0.660 to 0.882) across several cancer types and therapies [93]. Another study outlined a model including exercise physiology parameters, such as maximal oxygen uptake (VO2 max), to predict CTRCD; however, it was limited by a lack of validation cohorts [94]. Other multimodal models have incorporated imaging, serum and genetic variables [72]. Beyond improved predictive performance, these multimodal models better distinguish individuals during risk assessment, enabling individualised cardio-oncology care. This may improve the efficiency of cardio-oncology care and improve treatment outcomes by directing cardioprotective strategies to those at greater risk.

Many studies considering novel multimodal CTR-CVT risk scores are limited in several ways. As shown in Table 4, they often lack external validation, and when they include it, the validation cohort is sometimes small or is drawn from the same wider population as the training dataset. As a result, the external validation and training cohorts may share characteristics and common geographical factors, thus limiting applicability to broader populations. For example, one recent study used a time-based cut-off in the wider population to derive a validation cohort for CTRCD prediction in patients treated with anthracycline and trastuzumab [93]. Follow-up duration also varies greatly, with some risk models derived from datasets with only six months of follow-up and others with up to 20 years. When paired with large training and validation datasets, longer follow-up durations are likely to enable more detailed risk prediction.

Although the novel multimodal risk models cannot be compared directly due to methodological differences, the logistic regression model developed by Chen et al. [97] shows promise for predicting immune checkpoint inhibitor-related cardiovascular toxicity, given its high AUC and broad range of cardiovascular outcomes. (Table 4). The input features used are also readily available in routine clinical practice. However, the study was retrospective, with no external validation, so it remains unclear whether the model would perform similarly in other populations. The relatively small size of this cohort and other cohorts in Table 4 risks model overfitting, a phenomenon in which a prediction model performs well on the original training data but poorly on novel data. Brown et al. [98] addressed these shortcomings by deriving several machine learning-based risk models with strong predictive performance for post-cancer CVD in a large cohort with extensive follow-up and external validation. However, this study did not assess the calibration or clinical utility of the risk models, both of which are essential for real-world prediction model use alongside conventional discrimination measures (AUC). Conversely, Chen et al. [95] reported good discrimination, calibration and clinical utility of a separate XGBoost CTRCD risk prediction model, which suggests this model may be more useful than others. However, a cautious approach is needed when comparing risk model performance, as outcome definitions are often different.

Another potential drawback of complex multimodal risk models is the financial cost likely associated with large-scale implementation. Moreover, some input variables, such as transcriptomic data and certain advanced imaging variables, may not be readily available in clinical practice or may require close collaboration between a cancer care provider and a research facility. This limits the throughput and scalability of risk models to primary practice or resource-limited cancer centres. Additionally, other variables, such as VO2 max, may be technically infeasible to measure in patients with acute oncological disease. Future studies must balance the added predictive power of additional variables with measurement burden. Prospective evaluation is also needed to determine whether model-guided surveillance improves early detection, reduces unnecessary testing, prevents severe cardiovascular events and enables safer continuation of effective anticancer therapy without cardiovascular toxicity.

## 5. Digital Innovation

A growing evidence base suggests that digital tools may improve the prediction, detection and prevention of CTR-CVT [99]. Their value lies in an integrated model of care combining data and specialist judgement. Different machine learning methods can process structured variables, while multimodal models can integrate electronic health record data, imaging data and other clinical data [100]. These approaches may identify high-risk patients that conventional assessments miss. Dynamic models could update risk estimates throughout treatment and help assess the effectiveness of cardioprotective strategies [101]. Advanced machine learning algorithms, such as Extreme Gradient Boosting (XGBoost) and random forests, can be applied to multimodal data to predict CTR-CVT (Table 4). For non-linear data, these models can be more effective than logistic regression (LR) for classification and prediction tasks [102,103]. Indeed, Brown and colleagues found that an XGBoost model outperformed an LR model for predicting HF and CAD in cancer survivors [98]. Furthermore, Deep Learning (DL), an advanced machine learning technique that handles unstructured data through deep neural networks, may be useful for CTR-CVT prediction. A recent study suggested this, finding that a single-modality CMR-based deep learning CTR-CVT prediction model outperformed conventional multimodal machine learning methods that require human-led feature engineering and domain knowledge [96]. However, DL requires extremely large datasets and computational power, which may be difficult in resource-limited settings [104]. The environmental impact of this cannot be overlooked [105]. Notably, a non-cardio-oncology study found that a conventional machine learning Random Forest (RF) classifier performed similarly to a DL model for AF prediction from baseline ECGs [106]. In the same study, DL only outperformed RF for a separate regression task. Taken together, this suggests that the extra data and computational power required for DL techniques are only justifiable in specific settings where they provide additional predictive power. However, predictive performance does not always translate into clinical utility or clinically relevant outcomes, which net benefit analysis can assess [107]. Overfitting and underfitting also threaten model validity, describing cases where a model is too complex or too simple, respectively [108]. Dataset shift, in which data encountered in practice differ from training data, and data leakage, in which model development uses information unavailable at the time of prediction, can also undermine model validity [109,110]. Prospective validation of risk models in real-world cohorts and randomised controlled trials may mitigate these risks and are needed to test clinical utility, clinical feasibility and technical feasibility.

Digital innovation in health systems can aid CTR-CVT prediction [10]. For example, linked electronic health records (EHRs) could provide a longitudinal view of treatment and cardiovascular status by linking oncology notes, prescribing data, laboratory results and imaging [111]. This could enable implantation of clinical decision-support tools and risk scores. Natural language processing may extract symptoms and toxicities from unstructured records to train and improve risk prediction models [112]. This could be potentially formalised into dedicated pathways, such as the Explainable Holistic AI in Medicine (HAIM) framework, which is an experimental pathway that uses generative large language models to generate task-specific summaries from unstructured data, such as clinical notes, that can then be used as inputs in downstream predictive models [113]. Furthermore, digital innovation in clinical imaging analysis systems may improve CTR-CVT prediction by increasing the reproducibility of GLS and volumetric measurements, increasing analysis throughput, and identifying subtle abnormalities before overt cardiac dysfunction [114].

Other authors have proposed novel methods for risk prediction that incorporate AI models and wearable technology [115]. Wearable technology could extend surveillance beyond individual hospital visits, helping to identify trends that might otherwise be missed and improving access to monitoring where specialist services are limited. However, some abnormalities detected may not translate to clinically relevant CTR-CVT, and there is a lack of validated thresholds and escalation pathways, with many studies assessing technical performance rather than clinical outcomes [116].

In summary, digital innovation may support CTR-CVT risk stratification, increase productivity and improve coordination between oncology and cardiology [117]. However, several critical considerations remain. Firstly, the performance of digital tools depends on data quality, acquisition standards and the development population [118]. Poorly designed clinical decision support systems may also generate excessive alerts, so digital support must prioritise actionable information. False-positive alerts from wearable technology may cause patient anxiety, unnecessary testing and premature discontinuation of effective therapy [119]. Risk prediction models may perform less accurately in under-represented groups [120]. Furthermore, privacy, cybersecurity, informed consent, data stewardship and accountability for remote alerts require clear governance. These challenges are amplified in low- and middle-income countries, where access to specialist services, advanced testing and digital infrastructure may be limited [121]. Low-cost mobile systems, portable ECG technologies and teleconsultation may therefore be more practical than computationally intensive platforms [121]. Future research should prioritise prospective, multi-institutional studies, external validation and clinically meaningful outcomes.

## 6. Evidence Gaps and Future Directions

The evidence presented supports a multimodal approach to CTR-CVT prediction and assessment, but several evidence and implementation gaps remain that warrant future research. Most studies focus on predicting CTRCD and LVEF decline in the context of anthracycline or trastuzumab therapy, with little prospective external validation. It is unclear whether these findings generalise to other forms of CTR-CVT or to other anticancer therapies. Without prospective external validation, it is also unclear whether strong predictive performance leads to clinically meaningful outcomes, with sufficient time to initiate cardioprotective strategies before CTR-CVT develops. Regarding specific risk model input features, a recent study showed adding polygenic risk scores to clinical covariate models increased the predictive performance for CVD in women with a history of breast cancer [68]. The impact of adding other multimodal data, such as imaging data, remains unclear and requires investigation. As outlined above, deep learning and CMR radiomic features may be useful in this context.

There is currently limited evidence as to the optimal frameworks for implementing multimodal data for CTR-CVT risk prediction in routine clinical practice, as well as the best methods to collect data for model input features, particularly when datasets are large and unstructured [10]. We propose that the Explainable HAIM framework, or a similar pipeline, may be useful for predicting anticancer therapy-specific cardiovascular toxicity. This experimental framework incorporates demographic data, time-series data (including biomarker data), free text data (including imaging reports) and imaging data, which undergo processing and early fusion to generate embeddings that can be input into machine learning algorithms [113,122]. These frameworks have proven effective for predicting chest pathology using multimodal data but are relatively unexplored in the CTR-CVT prediction setting. Real-world clinical validation is also needed before implementation.

The impact of ethnicity and baseline CVD on the predictive performance of CTR-CVT prediction models remains uncertain, particularly as ethnic minority groups have often been underrepresented in cardio-oncology trials [123]. People with baseline CVD are also sometimes excluded from large oncology studies. The few existing studies considering the role of ethnicity have conflicting findings, with one study [68] suggesting inter-ethnic differences and another [124] suggesting no effect. Further validation studies are needed to characterise the algorithmic fairness of CTR-CVT prediction models. Finally, most studies and risk models are derived from Western European and North American populations, which limits the generalisability of findings to global cohorts and raises the issue of potential differences in predictive performance across populations. This is relevant as many novel predictive AI CVD risk models do not offer regional calibrations and are often not validated in global cohorts [125]. Similarly, other evidence suggests that CVD risk scores frequently perform differently in non-original cohorts [126]. For precision cardio-oncology to be a global field, global data registries are needed to ensure applicability, generalisability and transferability of science and care.

Digital twins are a growing area of research in cardiology and have been used to model cardiomyopathies and predict sudden cardiac death [127]. Beyond modelling doxorubicin-induced arrhythmia, digital twins have not been explored extensively in the setting of CTR-CVT [128]. Future research may uncover a role for digital twins in CTR-CVT risk prediction.

## 7. Conclusions

The existing evidence supports a multimodal genome–phenome approach to CTR-CVT risk stratification. Most evidence currently supports combined imaging and circulating biomarker-based strategies for anthracycline-induced CTRCD prediction. Still, promising evidence exists for other modalities and multi-omic data, including radiomics and genomics. Future work should consider the role of multimodal approaches in other forms of CTR-CVT, as well as broader applications in cardio-oncology, such as post-treatment surveillance.

## Figures and Tables

**Table 4:** A selection of novel multimodal, integrated genome–phenome models for cancer therapy-related cardiovascular toxicity (CTR-CVT) prediction and assessment.

Anticancer Therapies	Reference	Outcome(s)	Follow-Up Duration	Cohort Size	Algorithm	Variables Used in Multimodal Model	Model Performance	External Validation?
Models for predicting CTRCD								
Anthracyclines and trastuzumab	Chen et al. [95]	CTRCD ^1^	-	423 breast cancer patients	XGBoost	Clinical: age, AC + trastuzumab Echo: baseline LVEF < 60% ECG: baseline abnormality	AUC: 0.782 NPV: 0.860 PPV: 0.605	No
Anthracyclines, cyclophosphamide, vincristine, radiotherapy and immunotherapy	Bursacovschi et al. [94]	CTRCD at six months	6 months	127 non-Hodgkin lymphoma patients	LR	Clinical *: cumulative doxorubicin dose, comorbidities ECG *: SDNNi, mean heart rate/24 h, Mean QTc/24 h Echo *: LVEDV, LA volume Serum *: NT-pro BNP, haemoglobin	AUC: 0.950	No
Anthracyclines and trastuzumab	Yu et al. [96]	CTRCD ^2^ within 15 months post-anthracycline initiation	Up to 15 months	176 breast cancer patients	LR	Clinical and serum: HFA-ICOS risk score CMR: LVESV, LVEDV, LVEF, GLS	AUC: 0.710 Sensitivity: 0.750 Specificity: 0.560 F1 Score: 0.560	Yes
					XGBoost		AUC: 0.710 Sensitivity: 0.250 Specificity: 0.900 F1 Score: 0.320	
Anthracycline	Mahmoud et al. [72]	Deterioration in GLS > 15%	6 months	55 breast cancer patients	**-**	Echo: Δ GLS > 10% Serum: Hs-troponin I > 11 ng/L Genetic: Δ *TNNI3K* expression > 24%	AUC: 0.924 PPV: 1.000 NPV: 0.960	No
Models for predicting other CVD								
Radiation, Anthracyclines, Trastuzumab and other SACT	Zhou et al. [93]	CAD	0–10+ years	4309 oncology patients	LR	Clinical: comorbidities, age, signs and symptoms, SBP, DBP, HR, family history, smoking history, sex Echo: LVEF, LVESVi, ESV Serum: creatinine, lipids, potassium, sodium, AST, WBC, glucose	AUC: 0.821	Yes
		Atrial fibrillation					AUC: 0.787	
		Heart Failure					AUC: 0.882	
		Stroke					AUC: 0.660	
		MI					AUC: 0.807	
		CTRCD ^1^					AUC: 0.802	
Immune checkpoint inhibitors	Chen et al. [97]	Cardiomyopathy	Average 1.91 years	375 patients receiving ICI	-	CT: coronary artery calcium score > 10 U Serum: NT pro-BNP ≥ 125 pg/mL, cTNT ≥ 6 ng/L, hs-CRP ≥ 3 mL/L	AUC: 0.847	No
		HF/Myocarditis					AUC: 0.854	
		HF/Myocarditis/Death/Stroke/CAD/AF					AUC: 0.816	
Several therapies	Brown et al. [98]	De-novo post-cancer CVD	20 years	3835 cancer patients	RF	Clinical: age, peripheral artery disease Imaging: cardiomegaly, cardiomyopathy Serum: troponin-T	AUC: 0.802	Yes
					DT		AUC: 0.735	
					LR		AUC: 0.826	

AC anthracycline; AF atrial fibrillation; AST aspartate aminotransferase; AUC area under the curve; CAD coronary artery disease; CMR cardiovascular magnetic resonance; cTNT cardiac troponin T; CTRCD cancer therapy-related cardiac dysfunction; DBP diastolic blood pressure; DT decision tree; ESV end systolic volume; HR heart rate; hs-CRP high sensitivity C-reactive protein; ICI immune checkpoint-inhibitor; LR Logistic Regression; LVEDV left ventricular end-diastolic volume; LVEF left ventricular ejection fraction; LVESV(i) left ventricular end-systolic volume (index); MI myocardial infarction; NPV negative predictive value; NR not reported; PPV positive predictive value; RF Random Forest; SACT systemic anticancer therapy; SBP systolic blood pressure; SDNNI standard deviation of the normal-to-normal R-R intervals index; WBC white blood cells. CTRCD defined as: ^1^ LVEF decline by >10 points to value < 53%; ^2^ ≥10% reduction in LVEF from baseline, or ≥5% to an LVEF of <55% with heart failure symptoms. * only strongest predictors by SHAP (SHapley Additive exPlanations) analysis shown—additional variables include serum markers and echocardiographic markers.

## Data Availability

No new data were created or analyzed in this study. Data sharing is not applicable to this article.
